# Synthesis and secretion of transforming growth factor beta isoforms by primary cultures of human breast tumour fibroblasts in vitro and their modulation by tamoxifen.

**DOI:** 10.1038/bjc.1996.365

**Published:** 1996-08

**Authors:** J. R. Benson, L. M. Wakefield, M. Baum, A. A. Colletta

**Affiliations:** Hartwell Laboratory, Section of Academic Surgery, Royal Marsden Hospital, London, UK.

## Abstract

**Images:**


British Journal of Cancer (1996) 74, 352-358
© 1996 Stockton Press All rights reserved 0007-0920/96 $12.00

### Synthesis and secretion of transforming growth factor beta isoforms by primary cultures of human breast tumour fibroblasts *in vitro* and their modulation by tamoxifen

JR Benson[1], LM Wakefield[2], M Baum[1] and AA Colletta[1]

[1]*The Hartwell Laboratory, Section of Academic Surgery, Institute of Cancer Research, The Royal Marsden Hospital, Fulham Road, London SW3 6JJ, UK;* [2]*Laboratory of Chemoprevention, National Cancer Institute, Bethesda, MD 20892, USA.*

**Summary** Tamoxifen may mediate its effect in early breast cancer in part via an oestrogen receptor (ER)-independent pathway by directly stimulating fibroblasts to produce the negative paracrine growth factor transforming growth factor (TGF)-$\beta$. We have previously shown that secretion of this factor is induced 3-to 30-fold in human fetal fibroblasts *in vitro*, and by stromal fibroblasts *in vivo* following tamoxifen treatment of ER-positive and ER-negative breast cancer patients. Primary cultures of breast tumour fibroblasts have been exposed to tamoxifen for 48 h, and rates of secretion of TGF-$\beta_1$ and TGF-$\beta_2$ measured using a quantitative immunoassay. Fibroblast strains derived from malignant and benign tumours produced and secreted similar amounts of TGF-$\beta_1$, but benign breast tumour fibroblasts secreted significantly higher levels of TGF-$\beta_2$ compared with fibroblasts of malignant origin. Tamoxifen did not induce any consistent increase in TGF-$\beta$ secretion into the conditioned medium, but immunofluorescence analysis for the intracellular form of TGF-$\beta_1$ revealed evidence of increased immunoreactive protein in tamoxifen-treated fibroblasts, which is localised to the nucleus. Therefore synthesis of TGF-$\beta_1$ appears to be stimulated by tamoxifen, but increased secretion may be abrogated *in vitro*. Furthermore, using immunocytochemistry and transient transfection with an ER-responsive reporter construct, no ER was demonstrable in these fibroblasts supporting the proposed ER-independent paracrine pathway.

**Keywords:** breast cancer; growth factor; paracrine mechanism; tamoxifen

Initial results from the Nolvadex Adjuvant Trial Organisation (NATO) (1988) and Medical Research Council Scottish trials (1987) revealed that the efficacy of adjuvant tamoxifen in early breast cancer appeared to be independent of oestrogen receptor (ER) status. In the 8 year analysis of the NATO trial, division of patients according to ER status did not eliminate the favourable effects of tamoxifen treatment, and multivariate regression analysis did not show any significant difference in treatment effect between ER-positive and ER-negative patients. The more recent overview by the Early Breast Cancer Trialists Collaborative Group (1992) confirmed that ER status fails to select a group of patients who will not benefit from adjuvant tamoxifen therapy, and in particular reaffirmed that ER-negative patients obtain unequivocal benefit.

These counterintuitive results are difficult to reconcile with the classical mode of action of anti-oestrogens as competitive antagonists for the ligand binding site of the ER, a mechanism that is precluded in ER-negative cells (Terenius, 1968). It was therefore proposed that tamoxifen directly stimulates fibroblasts to produce and secrete negative growth modulators, which act upon neighbouring malignant epithelial cells in a negative paracrine manner (Colletta *et al.*, 1990). Certain other observations contributed to formulation of this hypothesis; firstly, the timing of androgen receptor expression in the mesenchyme of the developing rodent prostate–androgen receptors are expressed on the mesenchyme of hormonally sensitive tissues before their expression of epithelial cells, implying that hormones can act indirectly upon epithelium (Cunha and Donjacour, 1987). Secondly, skin fibroblasts from patients with a family history of breast cancer display fetal-like characteristics, thus alluding to some systemic abnormality of fibroblasts (Haggie *et al.*, 1987). Finally desmoids, which are pure mesenchymal tumours, undergo dramatic clinical response to tamoxifen and related triphenylethylenes, implying a direct effect of these agents upon fibroblasts (Brookes *et al.*, 1992).

There is now evidence for stromal induction of the negative growth modulator, transforming growth factor $\beta$ (TGF-$\beta$) both *in vitro* (Colletta *et al.*, 1990) and *in vivo* (Butta *et al.*, 1992) in response to tamoxifen. TGF-$\beta$ is a member of a superfamily of regulatory peptides, existing as three isoforms TGF-$\beta_1$, TGF-$\beta_2$ and TGF-$\beta_3$ in mammalian species. These are multifunctional peptides that are usually stimulatory to cells of mesenchymal origin (Roberts *et al.*, 1981), but inhibitory to certain epithelia (Roberts *et al.*, 1985). This growth factor is involved in cellular proliferation and differentiation during development, and defective TGF-$\beta$ signalling may be implicated in carcinogenesis (Roberts *et al.*, 1988). Specific roles for TGF-$\beta$ in malignant predisposition and progression have been proposed, owing either to loss of sensitivity to TGF-$\beta$ (Tucker *et al.*, 1984) or defective intrinsic production by stromal cells, leading to reduced negative paracrine influences upon neighbouring epithelial cells (Benson and Baum, 1993).

Previous *in vivo* studies have suggested that stromal fibroblasts are the source of extracellular TGF-$\beta_1$, which is up-regulated following primary tamoxifen therapy of ER-positive and ER-negative breast cance patients (Butta *et al.*, 1992). We have further investigated this concept and present evidence here that breast tumour fibroblasts are a rich source of TGF-$\beta_1$ and that synthesis of this negative growth factor can be modulated by tamoxifen, suggesting a mechanism whereby this agent could augment the negative paracrine regulation of epithelial proliferation by fibroblasts.

### Methods

### *Primary culture of fibroblasts*

Primary cultures of fibroblasts were derived from patients with either malignant (strains A–D) or benign (E–G) breast tumours. The clinical details of these patients are summarised in Table I. A further strain (H) was obtained from a skin

Correspondence: JR Benson
Received 27 October 1995; revised 4 March 1996; accepted 5 March 1996

**Table I** Clinicopathological features of patients from whose breast tumours fibroblast strains were derived

| *Cell strain* | *Patient age (years)* | *Pathology* | *Grade* | *Nodal status* | *ER status* |
|---|---|---|---|---|---|
| A | 64 | IDC (NOS) | PD (III) | Unknown[a] | Unknown |
| B | 61 | IDC (NOS) | MD (II) | Negative | Negative |
| C | 55 | IDC (NOS) | PD (III) | Negative | Positive |
| D | 73 | IDC (NOS) | MD (II) | Positive | Negative |
| E | 51 | FA | – | – | – |
| F | 38 | FA | – | – | – |
| G | 60 | FC | – | – | – |
| H | 60 | Skin | – | – | – |

[a]No axillary surgery. IDC, infiltrating ductal carcinoma; NOS, type 'not otherwise specified', MD, moderately differentiated; PD; poorly differentiated, FA, fibroadenoma; FC, fibrocystic disease.

sample of a patient with benign breast disease, and these were considered to be normal skin fibroblasts. Specimens of tissue, collected at surgery, were washed immediately in RPMI-1640 medium following collection and excess fat trimmed off. Specimens were minced into small fragments (2–3 mm) and digested overnight with collagenase [type IIS (Sigma) 1 μg $ml^{-1}$] for a period of 24 h. The resulting cell suspension was centrifuged (580 *g*, 10 min) and the pellet was resuspended in complete medium, consisting of minimal essential medium (MEM) supplemented with 10% fetal calf serum (FCS), basic fibroblast growth factor (FGF) (25 ng $ml^{-1}$), L-glutamine (2 mM), penicillin (1000 units $ml^{-1}$), streptomycin (100 μg $ml^{-1}$) and fungizone (25 ng $ml^{-1}$) (Freshney, 1987). Flasks were incubated at 37°C, 100% humidity and 5% carbon dioxide. Cultures were left undisturbed for 3–4 days, after which time fibroblasts were seen to be attaching. Cells were passaged when cultures reached confluence after 10–14 days, and fibroblasts used between passages 5–10.

*Verification of fibroblast origin*

The identity of fibroblasts was confirmed not only by their characteristic morphology, but also by staining for the specific immunophenotype. Fibroblasts were seeded into slide flasks at a density of $10^5$ cells $ml^{-1}$ and grown to 80–90% confluence, followed by fixation in ice-cold acetone. Following blocking of non-specific binding sites with 50% (v/v) porcine serum in phosphate-buffered saline (PBS), cells were labelled with 5 μg $ml^{-1}$ of an anti-vimentin mouse primary antibody (Boehringer-Mannheim) and identified with 20 μg $ml^{-1}$ of a secondary anti-mouse antibody conjugated to rhodamine. To verify that cultures were free from epithelial contamination, cells were labelled with 20 μg $ml^{-1}$ of an anti-keratin primary antibody (ICN Immunobiologicals). Cells were photomicrographed using a BioRad MRC 600 Nikon confocal fluorescence microscope.

*Oestrogen receptor content of fibroblasts*

Transfection of fibroblasts–primary fibroblast strains were grown to 50–60% confluence in 90 mm petri dishes in phenol red-free improved minimal essential medium (IMEM), containing 10% FCS [dextran-coated charcoal (DCC) treated] (Green and Leake, 1987), FGF, L-glutamine, antibiotics and fungizone in the above concentrations. Cells were transiently transfected with the ER-responsive reporter construct ERE-tk-CAT, consisting of the oestrogen response element (ERE) (Kumar and Chambon, 1988) linked to a hymidine kinase promoter and the chloramphenicol acetyltransferase (CAT) reporter gene. Transfection was carried out using Lipofectin (Gibco BRL). As an internal control, cells were co-transfected with the pCH110 plasmid (Pharmacia), which contains the β-galactosidase gene. As a positive control, cells were in addition triple transfected with the human ER expression vector pSG5-HEO (hER) (Green *et al.*, 1986). Each fibroblast strain was transfected with the ERE-tk-CAT construct in the presence of either 10 nM oestradiol or 1 μM tamoxifen. Internal controls were transfected with the pCH110 and ERE-tk-CAT constructs in the absence of any ligand, and positive controls with both ERE-tk-CAT together with hER.

Aliquots of 10 μg of each type of DNA was added per dish, i.e. 20 μg in total except for the positive controls, which received an additional 10 μg of hER. Cells were exposed to the lipofectin/DNA complex in serum-free medium for 24 h, followed by complete medium (containing 10% DCC-treated serum) for a further period of 48 h, after which cells were harvested for functional assays. CAT activity was measured in cell extracts using [$^3$H]acetyl co-enzyme A (0.1 μCi) in a scintillation-based assay (Neumann *et al.*, 1987). CAT values were normalised to the β-galactosidase levels, which were measured spectrophotometrically (Sambrook *et al.*, 1989).

*Secretion of TGF-β isoforms into conditioned media of fibroblasts*

Fibroblasts were seeded into 25 $cm^2$ flasks in complete serum containing medium (MEM), and grown to subconfluence. Cells were washed twice with PBS and complete medium replaced with serum-free and phenol red-free medium supplemented with 5 μg $ml^{-1}$ bovine insulin and containing either 1 μM tamoxifen or ethanolic vehicle (0.1%, v/v). Conditioned media (CM) were collected after a period of 48 h into siliconised vessels, clarified by centrifugation and stored at −70°C before sandwich enzyme-linked immunosorbent assay (SELISA). Cell monolayers were trypsinised, and cells harvested for counting. For SELISA analysis 1 ml samples of CM were thawed and treated with a cocktail of protease inhibitors to yield 1 μg $ml^{-1}$ leupeptin, 1 μg $ml^{-1}$ aprotonin, 1 μg $ml^{-1}$ pepstatin, 120 μg $ml^{-1}$ phenylmethylsulphonyl fluoride (PMSF) and 100 μg $ml^{-1}$ bovine serum albumin.

Following precipitation of total protein from CM with 100% w/v trichloroacetic acid (TCA) protein pellets were washed with ether–ethanol (1:1, v/v) at 4°C and lyophilised before assay for TGF-$\beta_1$ and TGF-$\beta_2$ using highly specific SELISAS (Danielpour *et al.*, 1989; Flanders *et al.*, 1990).

*Staining of fibroblasts for the intracellular form of TGF-$\beta_1$*

Fibroblasts from breast tumours were grown in slide flasks to subconfluency in complete medium containing FCS. Cells were washed twice with PBS and 3 ml of serum-free and phenol red-free medium containing either 1 μM tamoxifen or ethanolic vehicle only added. After 48 h, media were discarded and cells fixed in ice-cold acetone. Non-specific binding sites were blocked with 50% (v/v) goat serum in PBS. Cells were exposed to primary polyclonal antibody to the intracellular form of TGF-$\beta_1$ (either anti-LC antibody raised against amino acids 1–30 of the mature TGF-$\beta_1$ or anti-precursor peptide raised against amino acids 266–278 of the TGF-$\beta_1$ precursor[23]) at 10 μg $ml^{-1}$ and incubated at 20°C for 2 h. Bound anti-TGF-$\beta_1$ antibody was identified using a secondary anti-rabbit IgG conjugated to the fluorescent marker Texas red. Cells were mounted in glycerol, containing 0.1% 1,4-phenyldiamine, and visualised with confocal microscopy.

### Results

*Verification of fibroblast origin*

Two fibroblast strains were stained with monoclonal antibodies to either vimentin or keratin. Figure 1a shows one strain (A) labelled with the anti-vimentin antibody. The majority of cells show strong immunoreactivity, whereas cells exposed to the secondary antibody show only background immunofluorescence (data not shown). Breast epithelial cells such as T47-D cells stain positively for the anti-keratin antibody, in contrast to cultures of fibroblasts where no significant staining of cells is seen (Figure 1b). This pattern of staining together with the characteristic spindle morphology of the cells confirms their fibroblast origin.

*Oestrogen receptor content of fibroblast*

Figure 2 shows the results of transiently transfecting four fibroblast strains derived from malignant breast tumours with an ER-responsive reporter construct. All four cell strains transfected with hER in the presence of its ligand (positive control, column 4) produce a high CAT signal, although the signal for one strain (D) is of relatively lower absolute value. In contrast, transfection of the ERE-tk-CAT construct without exogenous ER expression, either in the presence of $E_2$ (column 2) or tamoxifen (column 3), yields a signal similar to the vehicle control (column 1).

These results are consistent with absence of ER protein or other transactivating proteins that function at the ERE from breast tumour fibroblasts, and are in agreement with our immunohistochemical examination of these cells for ER, which shows no specific staining (data not shown). This is in accordance with our previous data for fetal lung and pituitary fibroblasts (Colletta *et al.*, 1990) and the data of others (Peterson *et al.*, 1987).

**Figure 1** (**a** and **b**) Verification of fibroblast origin with immunofluorescence. A representative strain of breast tumour fibroblasts stained with a monoclonal antibody against vimentin, a structural protein characteristic of cells of mesenchymal origin. (**a**) Cells stained with an anti-vimentin antibody conjugated to the fluorescent marker rhodamine. Strong cytoplasmic staining is seen, and cells have the typical spindle morphology of fibroblasts. (**b**) Cells stained with an anti-keratin antibody. The characteristic outline of fibroblasts is discernible, but minimal intracellular staining is seen.

*Secretion of TGF-β isoforms into conditioned media of fibroblasts*

The rates of secretion of TGF-$\beta_1$ and TGF-$\beta_2$ into the conditioned media of the eight fibroblast strains used in this study are shown in Table II. These values are the mean rates of secretion calculated for duplicate samples each based on either three (TGF-$\beta_1$) or two (TGF-$\beta_2$) determinations, with a lower limit of detection of 0.5 pM. All cell strains derived

**Figure 2** Measurement of ER content of breast tumour fibroblasts using transient transfection of an ER-tk-CAT reporter construct. Four strains of fibroblasts were transfected with the ERE-tk-CAT construct using Lipofectin. Cells were transfected either in the presence of E2 or tamoxifen. As an internal control, all cells were co-transfected with the pCH110 vector, and as a positive control, cells were in addition triple transfected with the hER construct. Cells tranfected with both ERE-tk-CAT and hER yielded a high CAT signal (column 4), in contrast to cells transfected with ERE-tk-CAT alone, either in the presence of $E_2$ (column 2) or tamoxifen (column 3), when CAT signals were similar to the internal controls (column 1). ▨, pCH110+ERE-tk-CAT; □, pCH110+ERE-tk-CAT+$E_2$; ■, pCH110+ERE-tk-CAT+tamoxifen; ▧, pCH110+ERE-tk-CAT+hER+$E_2$.

**Table II** Secretion rates of TGF-$\beta_1$ and TGF-$\beta_2$ into conditioned media of fibroblasts in presence and absence of tamoxifen

| Cell strain | *TGF-$\beta_1$* Control | *TGF-$\beta_1$* Tamoxifen | *TGF-$\beta_2$* Control | *TGF-$\beta_2$* Tamoxifen |
|---|---|---|---|---|
| | *(ng $10^6$ 48 $h^{-1}$)* | | | |
| A (malignant) | 3.91 ± 0.23 | 5.19 ± 0.50 | 0.08 ± 0.00 | 0.11 ± 0.00 |
| B (malignant) | 1.16 ± 0.13 | 1.04 ± 0.07 | 0.07 ± 0.01 | 0.05 ± 0.00 |
| C (malignant) | 3.09 ± 0.35 | 2.83 ± 0.18 | 0.07 ± 0.00 | 0.07 ± 0.00 |
| D (malignant) | 0.84 ± 0.07 | 1.03 ± 0.04 | < 0.01 | < 0.01 |
| E (benign) | 2.10 ± 0.00 | 2.68 ± 0.00 | 0.156 ± 0.02 | 0.170 ± 0.00 |
| F (benign) | 1.06 ± 0.00 | 0.78 ± 0.25 | 0.150 ± 0.00 | < 0.05 |
| G (benign) | 1.28 ± 0.00 | 1.10 ± 0.23 | 1.26 ± 0.13 | 1.35 ± 0.06 |
| H (skin) | 0.54 ± 0.00 | 0.57 ± 0.00 | < 0.05 | < 0.05 |

Fibroblasts derived from malignant (strains A – D) or benign (strains E – F) breast tumours, together with a single strain of skin fibroblasts (H) were grown to subconfluence in slide flasks and subsequently treated with either 1 μM tamoxifen or ethanolic vehicle for a period of 48 h. Conditioned medium was harvested and total protein precipitated with 100% (w/v) trichloroacetic acid before measurement of TGF-$\beta_1$ and TGF-$\beta_2$ levels using a sandwich ELISA. Each value is the mean rate of secretion calculated for duplicate samples each based on three (TGF-$\beta_1$) or two (TGF-$\beta_2$) determinations (lower limit of detection 1 pM concentration in sample of conditioned medium).

from malignant tumours (A–D) produce and secrete relatively large amounts of TFG-$\beta_1$ ranging from 0.84 to 3.91 ng $10^{-6}$ cells 48 $h^{-1}$, with a mean value of 2.25 ng $10^{-6}$ cells 48 $h^{-1}$. Although absolute levels of TGF-$\beta_1$ vary between fibroblast strains (up to 6-fold), levels for each strain are concordant with small standard deviations. Fibroblasts derived from benign tumours (E–G) secrete slightly lower levels of TGF-$\beta_1$, ranging from 1.06 to 2.10 ng $10^{-6}$ cells 48 $h^{-1}$ with a mean value of 1.48 ng $10^{-6}$ cells 48 $h^{-1}$. Levels of TGF-$\beta_1$ secretion are not statistically significantly different between fibroblasts derived from malignant *vs* benign breast tumour fibroblasts. Absolute levels of secretion by these breast tumour fibroblasts are higher than for normal skin fibroblasts (H). Levels of secretion of the $\beta_2$ isoform by fibroblasts derived from malignant tumours (A–D) are approximately 50-fold lower than those of TGF-$\beta_1$, but fibroblasts from benign tumours secrete significantly higher levels of TGF-$\beta_2$ that for one cell strain (G) approaches that of TGF-$\beta_1$. There is a statistically significant difference in levels of secretion of TGF-$\beta_2$ between benign and malignant breast tumour fibrobasts ($P<0.05$).

In contrast to fetal fibroblasts, tamoxifen does not induce any consistent increase in levels of TGF-$\beta_1$, although there is relatively modest increase in secretion of approximately 30% by strain A in response to tamoxifen. Although levels of secretion are not generally enhanced by tamoxifen, absolute rates are 3 to 4-fold higher than basal unstimulated values for fetal fibroblasts (0.4–0.6 ng $10^{-6}$ cells 48 $h^{-1}$, mean 0.5 ng $10^{-6}$ cells 48 $h^{-1}$) (Colletta *et al.*, 1990).

### *Immunofluorescence of fibroblasts for the intracellular form of TGF-$\beta_1$*

To further investigate TGF-$\beta$ synthesis in these cells, we employed immunofluorescence microscopy to examine the intracellular distribution and/or processing of TGF-$\beta$ after tamoxifen treatment. Figure 3 shows fibroblasts from a malignant breast tumour stained with the anti-LC (1–30) antibody, which specifically reacts with the intracellular form of TGF-$\beta_1$ (Flanders *et al.*, 1989), detected with a fluorescent secondary antibody. Cells in Figure 3a were treated with vehicle alone, whereas those in Figure 3b were treated with 1 $\mu$M tamoxifen for 48 h before staining. Immunofluorescence analysis yields a distinctive pattern of staining in the nuclear region, and tamoxifen treatment of fibroblasts dramatically increases the intensity of this immunofluorescence (Figure 3b). This response was observed in all strains of breast tumour fibroblasts tested, together with A549 cells, which are known to be a rich source of TGF-$\beta_1$ (Flanders *et al.*, 1989). Optical sectioning experiments in which confocal images are taken sequentially through the image plane indicate that this staining is indeed nuclear, and not confined to a peri-nuclear structure (Figure 4). To further confirm that the observed staining is attributable to TGF-$\beta_1$, the same fibroblasts have been stained with an antibody raised to the precursor region of TGF-$\beta_1$ (Flanders *et al.*, 1989). This antibody gives a broadly similar pattern of staining, and in particular there is a marked increase in intensity of nuclear staining following tamoxifen treatment (Figures 5a and b). Taken together, these data illustrate that tamoxifen treatment of primary breast cancer fibroblasts increases the extent of TGF-$\beta_1$ immunoreactivity but, unusually, this staining appears largely confined to the nucleus.

**Figure 3** (**a**–**c**) Staining of breast tumour fibroblasts for the intracellular (LC) form of TGF-$\beta_1$. Fibroblasts were grown to subconfluence in slide flasks and exposed to either tamoxifen or vehicle for 48 h before staining with the anti-LC antibody to the intracellular form of TGF-$\beta_1$. This primary antibody was detected with a secondary antibody conjugated to the immunofluorescent marker Texas red. (**a**) Cells treated with ethanolic vehicle followed by staining with anti-LC antibody. (**b**) Cells treated with tamoxifen (1000 nM) for 48 h before staining with anti-LC antibody. (**c**) Cells stained with rabbit IgG only as a primary antibody control. Staining for TGF-$\beta_1$ was predominantly distributed in the region of the nucleus, with no nuclear staining being seen with the control (rabbit IgG) antibody. This nuclear staining is dramatically increased following tamoxifen treatment of cells (**b**).

### Discussion

Recent *in vivo* studies demonstrating induction of extracellular TGF-$\beta_1$ by tamoxifen in both ER-positive and ER-negative patients provide strong evidence in support of negative paracrine regulation of breast cancer. Increased immunoreactive TGF-$\beta_1$ was observed between and around stromal cells with little increase in the vicinity of epithelial cells. Moreover, staining for the intracellular form of the peptide was largely confined to stromal cells (Butta *et al.*, 1992). These findings are consistent with the hypothesis that stromal fibroblasts are directly stimulated to produce and secrete a negative growth modulator that acts upon neighbouring epithelial cells in a paracrine manner. The data also suggest a potential role for the extracellular matrix in 'recruiting' newly synthesised TGF-$\beta_1$ adjacent to the tumour epithelium.

The preservation of tissue organisation in these immunohistochemical studies is a great advantage over *in vitro* studies

**Figure 4** Intracellular localisation of TGF-$\beta_1$ immunofluorescent staining of fibroblasts using confocal microscopy in which optical sections of 0.1 $\mu$m thickness were taken sequentially through the image plane at 1 $\mu$m intervals. Immunofluorescence is maximal in the central portion of the cell nucleus, and this confirms that staining is nuclear and not perinuclear.

**Figure 5** (**a** and **b**) Further characterisation of intracellular staining for TGF-$\beta_1$ using an antibody to the precursor region of TGF-$\beta_1$ (amino acids 266–278). This primary antibody was also detected with a secondary antibody conjugated to the immunofluorescent marker Texas red. (**a**) Cells treated with ethanolic vehicle followed by staining with precursor antibody. (**b**) Cells treated with tamoxifen (1000 nM) for 48 h before staining with precursor antibody. This antibody to the precursor region of TGF-$\beta_1$ demonstrates an essentially similar pattern of staining to the anti-LC antibody, although there appears to be greater cytoplasmic staining. Following exposure to tamoxifen, this nuclear staining is markedly increased (**b**) compared with control cells (**a**), and this co-localisation confirms that this staining is attributable to TGF-$\beta_1$.

on isolated cellular components. However, only an *in vitro* system can unequivocally demonstrate secretion of a substance by a particular cell type. The *in vitro* experiments reported here show that fibroblasts derived from both benign and malignant breast tumours produce and secrete relatively high levels of TGF-$\beta_1$. There is no statistically significant difference in levels of TGF-$\beta_1$ secretion between malignant and benign breast tumour fibroblasts, but the latter produce significantly higher levels of TGF-$\beta_2$ ($P<0.05$). This may indicate that differential quantitative expression of TGF-$\beta$ isoforms is important during neoplastic development. Absolute levels of TGF-$\beta_1$ are on average 3-to 4-fold higher than baseline unstimulated levels in fetal fibroblasts in which maximal induction ranged from 3-to 30-fold (Colletta *et al.*, 1990). However, contrary to any anticipation based on previous *in vitro* (Colletta *et al.*, 1990) and *in vivo* (Butta *et al.*, 1992) studies, tamoxifen did not induce any consistent increase in secreted levels of TGF-$\beta_1$ from these isolated breast tumour fibroblasts. Only one fibroblast strain (A) derived from a malignant tumour showed a modest increase in TGF-$\beta_1$ in response to tamoxifen.

Tamoxifen may increase the synthesis of TGF-$\beta_1$ in these breast tumour fibroblasts, but because of their isolation from neighbouring malignant epithelial cells *in vitro*, any increased secretion secondary to enhanced production of TGF-$\beta_1$ is abrogated. This interpretation is supported by results of staining for the intracellular form of TGF-$\beta_1$, demonstrating increased synthesis of TGF-$\beta_1$ in response to tamoxifen. However, co-culture of breast tumour fibroblasts with ER-negative breast carcinoma cells (BT-20) in a monolayer system has failed to restore any significant secretory response by fibroblasts (our unpublished observations), and more sophisticated 3-D systems may be required to achieve this.

The immunofluorescence data in Figures 3 and 5 reveal that staining for both TGF-$\beta_1$ and the precursor peptide occurs predominantly in the nuclear region with much weaker cytoplasmic staining. This unusual, but provocative, finding suggests that TGF-$\beta$ has distinct intracellular localisations. Moreover, two discrete forms of intracellular TGF-$\beta_1$ may exist – a secreted and a nuclear form. Like many other growth factors, TGF-$\beta$ is considered to act classically by interaction with cell-surface receptors, and subsequent activation of intracellular transduction pathways. However, recent evidence challenges this as an exclusive phenomenon for mediating the action of certain growth factors (Cross and Dexter, 1991). Cells may produce growth factors and related proteins that are not only destined for secretion, but that may also be diverted to the nucleus where they can directly influence nuclear events independently of any cognate receptor. The *int-2* gene appears to encode two similar products, but with different subcellular fates. One protein enters the secretory pathway, whereas an N-terminally extended protein is diverted to the nucleus

(Acland *et al*., 1990). Other examples of nuclear localisation of common growth factors such as epidermal growth factor and colony-stimulating factor 1 have been reported (Johnson *et al*., 1980; Yeh *et al*., 1987; Scholl *et al*., 1994).

Therefore, fibroblasts may produce two forms of TGF-$\beta_1$, one of which is destined for secretion and will act extracellularly via membrane receptors, and a second form that is localised/translocated to the nucleus. These secretory and nuclear forms of TGF-$\beta_1$ could be differentially induced by tamoxifen. Relative amounts of the secretory form of TGF-$\beta_1$ may be increased by tamoxifen *in vivo* (Butta *et al*., 1992), but isolated fibroblasts *in vitro* may respond aberrantly to tamoxifen with increased amounts of the nuclear form. Attempts to determine the molecular mass of the cytoplasmic and nuclear forms of TGF-$\beta_1$ by immunoblotting using the above antibodies were unsuccessful.

Control of the relative amounts of secretory and nuclear forms of TGF-$\beta_1$ may occur at the level of translation of the TGF-$\beta_1$ mRNA molecule. By analogy with the prostatic protein, probasin (Spence *et al*., 1989), both nuclear and secretory forms of TGF-$\beta_1$ could be encoded by a single mRNA molecule as a second 'in-phase' initiation codon exists within the coding region of the TGF-$\beta_1$ mRNA molecule that has a better sequence context for translational initiation (Derynck *et al*., 1985). Initiation from the downstream AUG would result in a smaller TGF-$\beta$ precursor (354 amino-acids) and eliminate the signal peptide sequence that might allow trafficking to intracellular locations such as the nucleus.

This increased intracellular staining for TGF-$\beta_1$ following tamoxifen treatment was also found in cells stained with anti-LC antibody and a secondary biotinylated antibody linked to a peroxidase-labelled avidin–biotin system. Furthermore, this response was observed in fibroblasts derived from both benign and malignant breast tumours, but not normal skin fibroblasts (data not shown). These findings suggest that fibroblasts from both benign and malignant tumours may display phenotypic features that are not shared by other somatic fibroblasts, and may be acquired during the process of neoplastic development. That phenotypic differences may exist between breast tumour fibroblasts and 'normal' fibroblasts is supported by the findings that conditioned media from benign and malignant breast tumours is stimulatory to MCF-7 cells *in vitro*, whereas media from normal skin fibroblasts is inhibitory to these cells (Adams *et al*., 1988). Aberrant stromal phenotypes in breast tumours may lead to deranged stromal–epithelial interactions and promote neoplastic progression.

Increased synthesis of TGF-$\beta_1$ is not associated with any concomitant elevation of mRNA levels in MCF-7 breast cancer cells (Knabbe *et al*., 1987) or fetal fibroblasts (Colletta *et al*., 1990). We have similar data from breast tumour fibroblasts *in vitro* (data not shown), and tamoxifen would therefore appear to enhance synthesis at a post-transcriptional level, although transcriptional mechanisms may also be operative depending on the local tissue levels of tamoxifen (Perry *et al*., 1995; Benson and Baum, 1996) and TGF-$\beta$ isoform type (Arrick *et al*., 1994; MacCallum *et al*., 1994).

The results of these *in vitro* investigations corroborate previous *in vivo* studies demonstrating induction of stromal TGF-$\beta_1$ by tamoxifen. In particular, they confirm that breast tumour fibroblasts are a potential source of TGF-$\beta_1$, and despite limitations of *in vitro* data, evidence is presented for a direct effect of tamoxifen upon tumour fibroblasts in the absence of measurable oestrogen receptor. However, such induction of TGF-$\beta_1$ may not be a property unique to tamoxifen. Recently, up-regulation of extracellular TGF-$\beta$ has been observed in prostate cancer patients following various forms of ablative androgen therapy (Muir *et al*., 1994). Therefore, TGF-$\beta$ induction may be a common step in several therapeutic interventions which may not involve classical hormone receptors.

The challenge for the future is to develop agents that can modulate fibroblast behaviour, and are of a specificity and potency that renders them clinically efficacious. Such a strategy may be especially pertinent in a chemopreventive setting and in early-stage malignancies in which tumour burden is modest and cells still possess appropriate receptors for negative growth modulators such as TGF-$\beta$.

**Acknowledgements**
The authors gratefully acknowledge the Cancer Research Campaign and the Committee for Clinical Research for supporting this work. We thank Pierre Chambon and Malcolm Parker for the gift of plasmids used in this study.

### References

ACLAND P, DIXON M, PETERS G AND DICKSON C. (1990). Subcellular fate of the Int-2 oncoprotein is determined by choice of initiation codon. *Nature*, **343**, 662–665.

ADAMS EF, NEWTON CJ, BRAUNSBERG H, SHAIKH N, GHILCHIK M AND JAMES VHT. (1988). Effects of human breast fibroblasts on growth and 17$\beta$ oestradiol dehydrogenase activity of MCF-7 cells in culture. *Breast Cancer Res. Treat*., **11**, 165–172.

ARRIK BA, GRENDELL RL AND GRIFFIN LA. (1994). Enhanced translational efficiency of a novel transforming growth factor beta 3 mRNA in human breast cancer cells. *Mol. Cell. Biol*., **14**, 619–628.

BENSON JR AND BAUM M. (1993). Breast cancer, desmoid tumours and familial adenomatous polyposis – a unifying hypothesis. *Lancet*, **342**, 848–850.

BENSON JR AND BAUM M. (1996). Tamoxifen-induced transforming growth factor $\beta$1 expression in human breast cancer cells (letter to the Editor). *Br. J. Cancer*, (in press).

BROOKES MD, EBBS SR, COLLETTA AA AND BAUM M. (1992). Desmoids treated with triphenylethylenes. *Eur. J. Cancer*, **28**, 1014–1018.

BUTTA A, MACLENNON K, FLANDERS KC, SACKS NPM, SMITH I, MACKINNA A, DOWSETT M, WAKEFIELD LM, SPORN MB, BAUM M AND COLLETTA AA. (1992). Induction of transforming growth factor beta$_1$ in human breast cancer *in vivo* following tamoxifen treatment. *Cancer Res*., **52**, 4261–4264.

COLLETTA AA, WAKEFIELD LM, HOWELL FV, ROOZENDAAL KEP, DANIELPOUR D, EBBS SR, SPORN MB AND BAUM M. (1990). Anti-oestrogens induce the secretion of active transforming growth factor beta from human fetal fibroblasts. *Br. J. Cancer*, **62**, 405–409.

CROSS M AND DEXTER TM. (1991). Growth factors in development, transformation and tumorigenesis. *Cell*, **64**, 271–280.

CUNHA GR AND DONJACOUR A. (1987). Stromal-epithelial interactions in normal and abnormal prostatic development. *Prog. Clin. Biol. Res*., **239**, 251–272.

DANIELPOUR D, DART LL, FLANDERS KC, ROBERTS AB AND SPORN M. (1989). Immunodetection and quantitation of the two forms of transforming growth factor $\beta$ secreted by cells in culture. *J. Cell Physiol*., **138**, 79–86.

DERYNCK R, JARRETT JA, ELLSON YC, EATON DH, BELL JR, ASSOIAN RK, ROBERTS AB, SPORN M AND GOEDDELL DV. (1985). Human transforming growth factor beta complementary DNA sequence and expression in normal and transformed cells. *Nature*, **316**, 701–705.

EARLY BREAST CANCER TRIALISTS COLLABORATIVE GROUP. (1992). Systemic treatment of early breast cancer by hormonal, cytotoxic or immune therapy. 133 randomised trials involving 31,000 recurrences and 24,000 deaths among 75,000 women. *Lancet*, **339**, 1–15 and 71–75.

FLANDERS KC, THOMPSON NL, CISSEL DS, VAN OBBERGEHN-SCHILLING E *et al.* (1989). Transforming growth factor β – histochemical localisation with antibodies to different epitopes. *J. Cell. Biol.*, **108**, 653–660.

FRESHNEY RI. (1987). Culture of Animal Cells – a Manual of Basic Technique. Alan Liss: New York.

GREEN S AND LEAKE R. (1987). Steroid Hormones – a Practical Approach. Green B and Leake R (eds). IRL Press: Oxford.

GREEN S, WALTER P, KUMAR V, KRUST A, BORNERT J-M, ARGOS P AND CHAMBON P. (1986). Human oestrogen receptor cDNA: sequence, expression and homology to v-*erb*-A. *Nature*, **320**, 137–139.

HAGGIE JA, SELLWOOD RA, HOWELL A, BIRCH JM AND SHOR SL. (1987). Fibroblasts from relatives of patients with hereditary breast cancer show foetal-like behaviour *vitro*. *Lancet*, **1**, 1455–1457.

JOHNSON LK, VLODAVSKY I, BAXTER JD AND GOSPODAROWICZ D. (1980). Nuclear accumulation of epidermal growth factor in cultured rat pituitary cells. *Nature*, **287**, 340–343.

KNABBE C, LIPPMAN ME, WAKEFIELD LM, FLANDERS KC, KASID A, DERYNCK R AND DICKSON RB. (1987). Evidence that transforming growth factor beta is a hormonally regulated negative growth factor in human breast cancer. *Cell*, **48**, 417–428.

KUMAR V AND CHAMBON P. (1988). The oestrogen receptor binds tightly to its responsive element as a ligand-induced homodimer. *Cell*, **55**, 145–156.

MEDICAL RESEARCH COUNCIL SCOTTISH TRIALS OFFICE. (1987). Adjuvant tamoxifen in the management of operable breast cancer. *Lancet*, **2**, 171–175.

MUIR G, BUTTA A, SHEARER RJ, FISHER C, DEARNLEY DP, FLANDERS KC, SPORN MB AND COLLETTA AA. (1994). Induction of TGF β in hormonally treated human prostate cancer. *Br. J. Cancer*, **69**, 130–134.

NEUMANN JR, MORENCY CA AND RUSSIAN KO. (1987). A novel rapid assay for chloramphenicol acetyltransferase gene expression. *Biotechniques*, **5**, 444.

NOLVADEX ADJUVANT TRIAL ORGANISATION. (1988). Controlled trial of tamoxifen as a single adjuvant agent in the management of early breast cancer. *Br. J. Cancer*, **57**, 608–612.

MACCALLUM J, BARTLETT JMS, THOMPSON AM, KEEN JC, DIXON JM AND MILLER WB. (1994). Expression of transforming growth factor β mRNA isoforms in human breast cancer. *Br. J. Cancer*, **69**, 1006–1009.

PERRY RR, KANG Y AND GREAVES BR. (1995). Relationship between tamoxifen-induced transforming growth factor β1 expression, cytostasis and apoptosis in human breast cancer cells. *Br. J. Cancer*, **72**, 1441–1446.

PETERSON DW, HOYER PE AND VAN DEURS B. (1987). Frequency and distribution of oestrogen receptor positive cells in normal non-lactating human breast tissue. *Cancer Res.*, **47**, 5748–5751.

ROBERTS AB, ANZANO MA, LAMB LC, SMITH JM AND SPORN MB. (1981). New class of transforming growth factors potentiated by epidermal growth factor: isolation from non-neoplastic tissues. *Proc. Natl Acad. Sci. USA*, **78**, 5339–5343.

ROBERTS AB, ANZANO MA, WAKEFIELD LM, ROCHE NS, STERN DF AND SPORN MB. (1985). Type β – transforming growth factor: a bifunctional regulator of cellular growth. *Proc. Natl Acad. Sci. USA*, **82**, 119–123.

ROBERTS AB, FLANDERS KC, KONDAIAH P, THOMPSON NL, VAN OBBERGEHN-SCHILLING E, WAKEFIELD L, ROSSI P, DE CROMBRUGGE B, HEINE E AND SPORN MB. (1988). Transforming growth factor β – biochemistry and roles in embryogenesis, tissue repair and remodelling, and carcinogenesis. *Rec. Prog. Horm. Res.*, **44**, 157–197.

SAMBROOK J, FRITSCH EF AND MANIATIS T. (1989). Molecular Cloning – a Laboratory manual, 2nd ed. Cold Spring Harbor Press: Cold Spring Harbor, NY.

SCHOLL SM AND POUILLART P. Is CSF-1 a key mediator in breast cancer invasion and metastasis? *The Lancet Conference*, Brugge, April 1994.

SPENCE AM, SHEPPARD PC, DAVIE R, MATUO Y, NISHI N, MCKEEHAN WL, DODD JG AND MATUSIK RJ. (1989). Regulation of a bifunctional mRNA results in synthesis of secreted and nuclear probasin. *Proc. Natl Acad. Sci. USA*, **86**, 7843–7847.

TERENIUS L. (1968). Two modes of interaction between oestrogen and anti-oestrogen. *Acta Endocrinol.*, **64**, 47–58.

TUCKER RF, BRANUM EL, SHIPLEY GD, RYAN RJ AND MOSES HL. (1984). Specific binding to cultured cells of $^{125}$I-labelled type β transforming growth factor from human platelets. *Proc. Natl Acad. Sci. USA*, **81**, 6757–6761.

YEH H-J, PIERCE GF AND DEUEL TF. (1987). Ultrastructural localisation of platelet-derived growth factor/v-sis related protein(s) in cytoplasm and nucleus of simian sarcoma virus-transformed cells. *Proc. Natl Acad. Sci. USA*, **84**, 2317–2321.


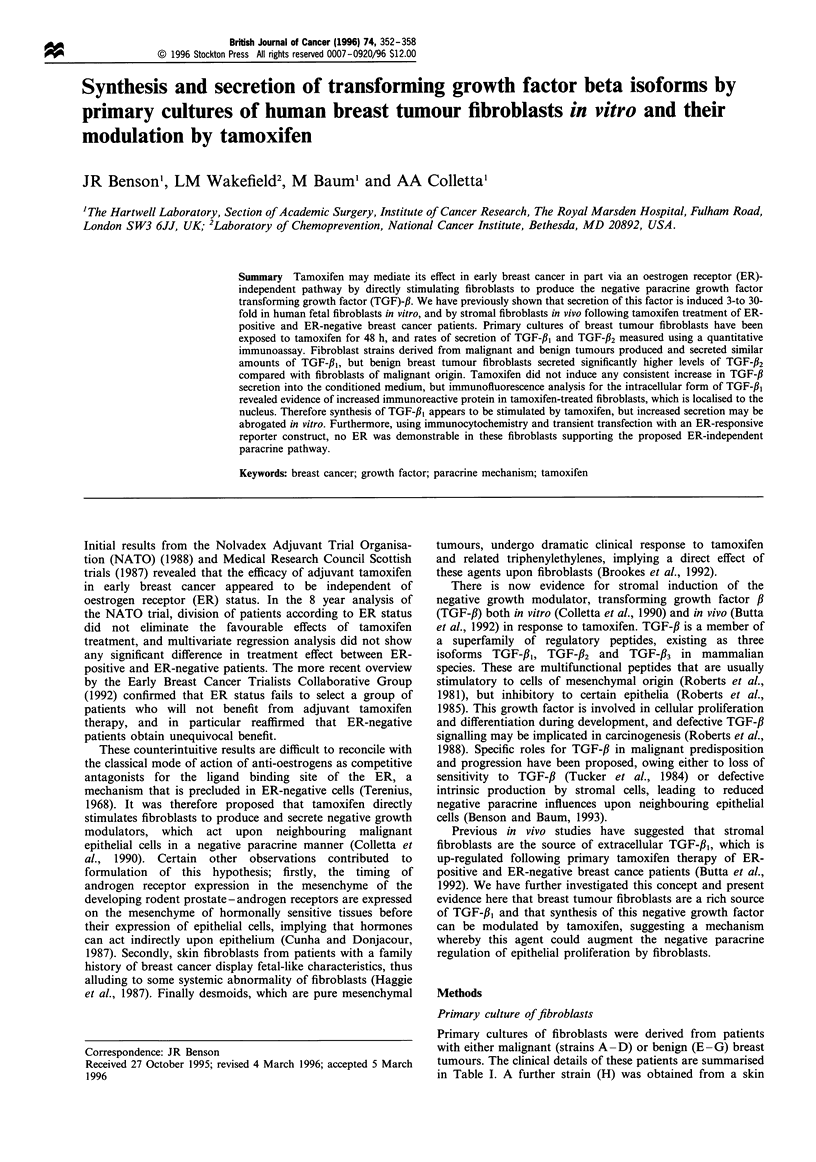

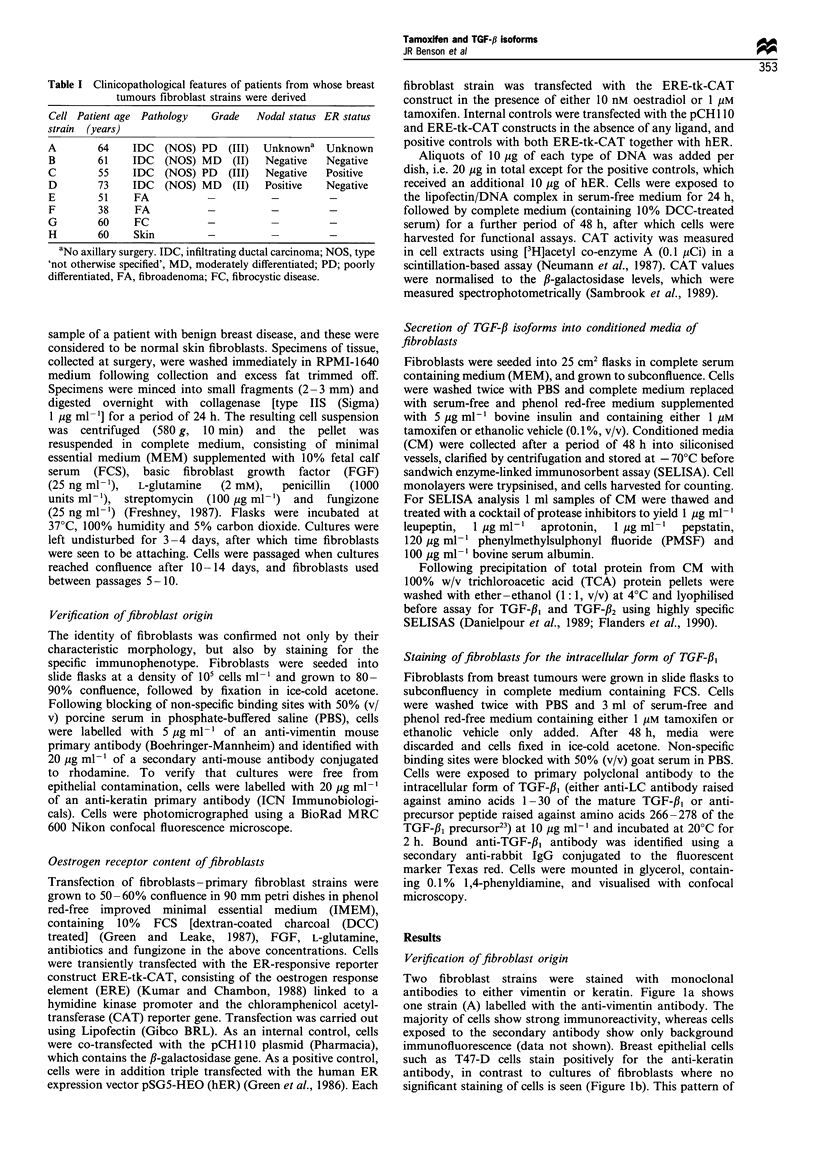

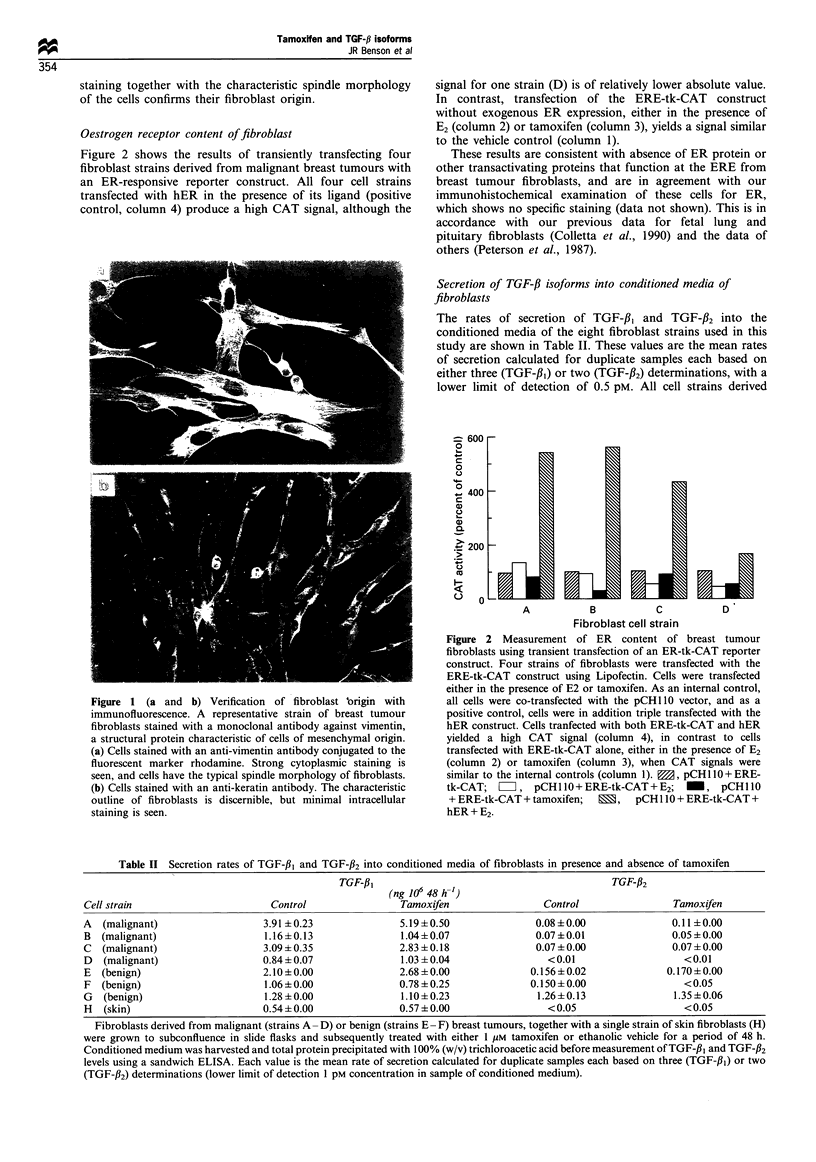

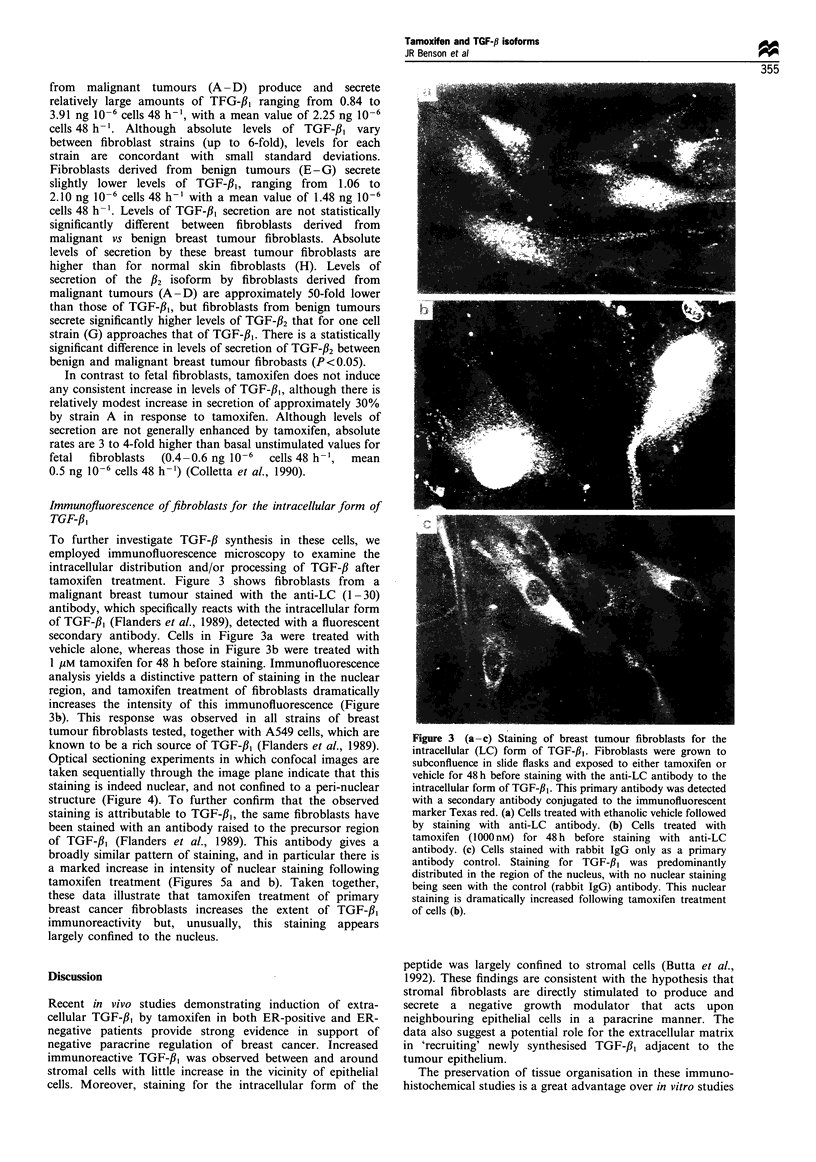

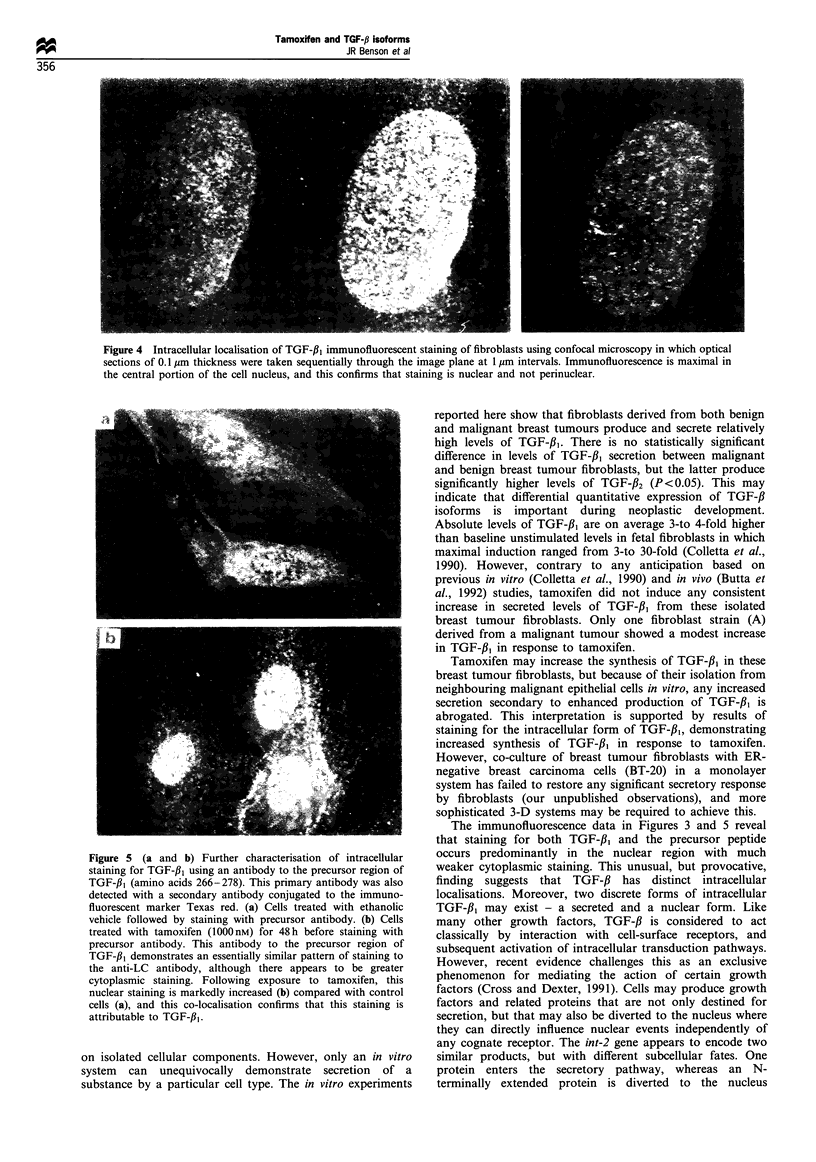

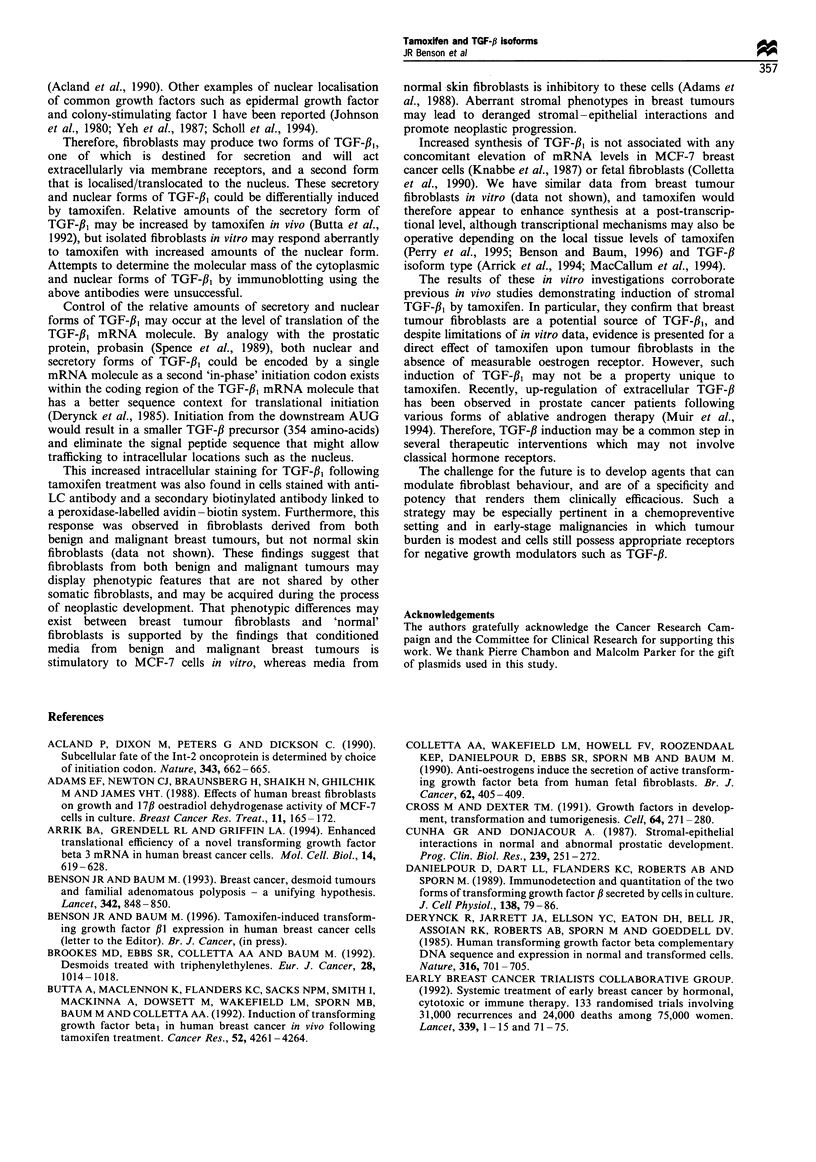

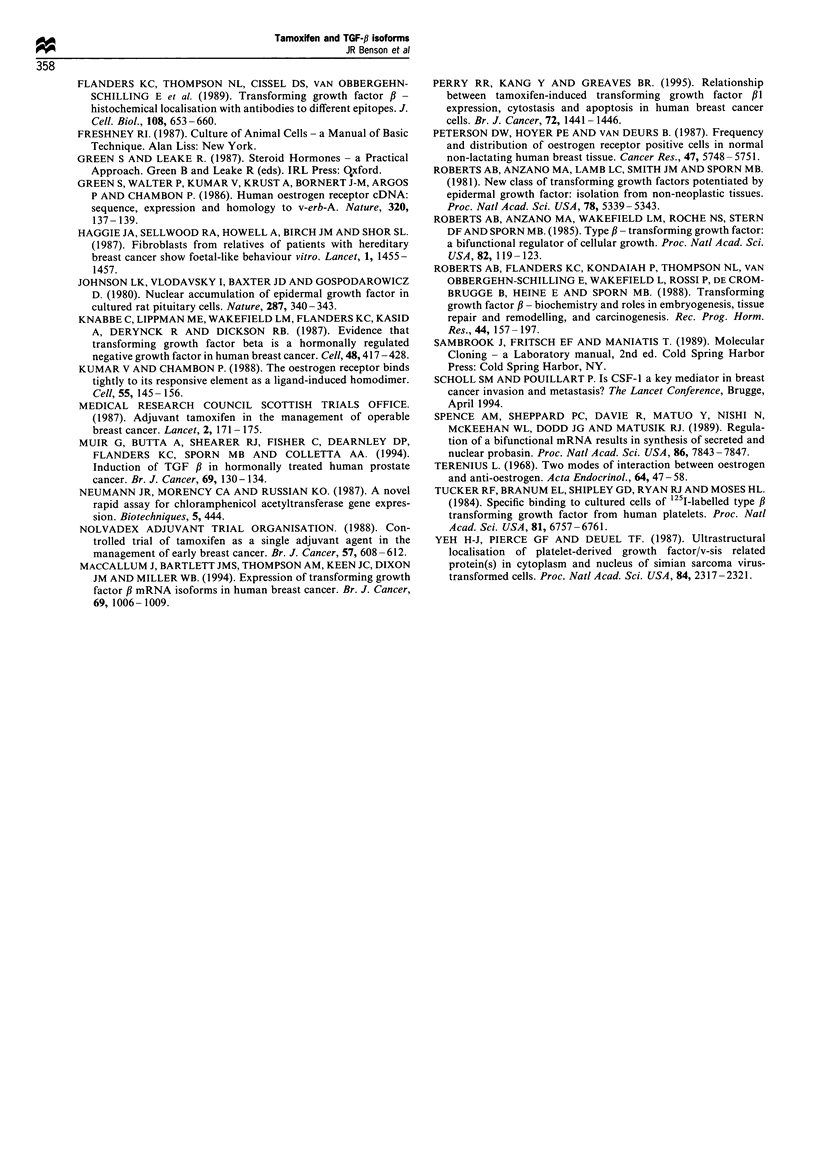


## References

[OCR_00698] Acland P., Dixon M., Peters G., Dickson C. (1990). Subcellular fate of the int-2 oncoprotein is determined by choice of initiation codon.. Nature.

[OCR_00706] Adams E. F., Newton C. J., Braunsberg H., Shaikh N., Ghilchik M., James V. H. (1988). Effects of human breast fibroblasts on growth and 17 beta-estradiol dehydrogenase activity of MCF-7 cells in culture.. Breast Cancer Res Treat.

[OCR_00709] Arrick B. A., Grendell R. L., Griffin L. A. (1994). Enhanced translational efficiency of a novel transforming growth factor beta 3 mRNA in human breast cancer cells.. Mol Cell Biol.

[OCR_00717] Benson J. R., Baum M. (1993). Breast cancer, desmoid tumours, and familial adenomatous polyposis--a unifying hypothesis.. Lancet.

[OCR_00727] Brooks M. D., Ebbs S. R., Colletta A. A., Baum M. (1992). Desmoid tumours treated with triphenylethylenes.. Eur J Cancer.

[OCR_00730] Butta A., MacLennan K., Flanders K. C., Sacks N. P., Smith I., McKinna A., Dowsett M., Wakefield L. M., Sporn M. B., Baum M. (1992). Induction of transforming growth factor beta 1 in human breast cancer in vivo following tamoxifen treatment.. Cancer Res.

[OCR_00737] Colletta A. A., Wakefield L. M., Howell F. V., van Roozendaal K. E., Danielpour D., Ebbs S. R., Sporn M. B., Baum M. (1990). Anti-oestrogens induce the secretion of active transforming growth factor beta from human fetal fibroblasts.. Br J Cancer.

[OCR_00746] Cross M., Dexter T. M. (1991). Growth factors in development, transformation, and tumorigenesis.. Cell.

[OCR_00750] Cunha G. R., Donjacour A. (1987). Stromal-epithelial interactions in normal and abnormal prostatic development.. Prog Clin Biol Res.

[OCR_00756] Danielpour D., Dart L. L., Flanders K. C., Roberts A. B., Sporn M. B. (1989). Immunodetection and quantitation of the two forms of transforming growth factor-beta (TGF-beta 1 and TGF-beta 2) secreted by cells in culture.. J Cell Physiol.

[OCR_00759] Derynck R., Jarrett J. A., Chen E. Y., Eaton D. H., Bell J. R., Assoian R. K., Roberts A. B., Sporn M. B., Goeddel D. V. (1985). Human transforming growth factor-beta complementary DNA sequence and expression in normal and transformed cells.. Nature.

[OCR_00779] Flanders K. C., Thompson N. L., Cissel D. S., Van Obberghen-Schilling E., Baker C. C., Kass M. E., Ellingsworth L. R., Roberts A. B., Sporn M. B. (1989). Transforming growth factor-beta 1: histochemical localization with antibodies to different epitopes.. J Cell Biol.

[OCR_00793] Green S., Walter P., Kumar V., Krust A., Bornert J. M., Argos P., Chambon P. (1986). Human oestrogen receptor cDNA: sequence, expression and homology to v-erb-A.. Nature.

[OCR_00801] Haggie J. A., Sellwood R. A., Howell A., Birch J. M., Schor S. L. (1987). Fibroblasts from relatives of patients with hereditary breast cancer show fetal-like behaviour in vitro.. Lancet.

[OCR_00807] Johnson L. K., Vlodavsky I., Baxter J. D., Gospodarowicz D. (1980). Nuclear accumulation of epidermal growth factor in cultured rat pituitary cells.. Nature.

[OCR_00813] Knabbe C., Lippman M. E., Wakefield L. M., Flanders K. C., Kasid A., Derynck R., Dickson R. B. (1987). Evidence that transforming growth factor-beta is a hormonally regulated negative growth factor in human breast cancer cells.. Cell.

[OCR_00815] Kumar V., Chambon P. (1988). The estrogen receptor binds tightly to its responsive element as a ligand-induced homodimer.. Cell.

[OCR_00843] MacCallum J., Bartlett J. M., Thompson A. M., Keen J. C., Dixon J. M., Miller W. R. (1994). Expression of transforming growth factor beta mRNA isoforms in human breast cancer.. Br J Cancer.

[OCR_00828] Muir G. H., Butta A., Shearer R. J., Fisher C., Dearnaley D. P., Flanders K. C., Sporn M. B., Colletta A. A. (1994). Induction of transforming growth factor beta in hormonally treated human prostate cancer.. Br J Cancer.

[OCR_00848] Perry R. R., Kang Y., Greaves B. R. (1995). Relationship between tamoxifen-induced transforming growth factor beta 1 expression, cytostasis and apoptosis in human breast cancer cells.. Br J Cancer.

[OCR_00854] Petersen O. W., Høyer P. E., van Deurs B. (1987). Frequency and distribution of estrogen receptor-positive cells in normal, nonlactating human breast tissue.. Cancer Res.

[OCR_00859] Roberts A. B., Anzano M. A., Lamb L. C., Smith J. M., Sporn M. B. (1981). New class of transforming growth factors potentiated by epidermal growth factor: isolation from non-neoplastic tissues.. Proc Natl Acad Sci U S A.

[OCR_00863] Roberts A. B., Anzano M. A., Wakefield L. M., Roche N. S., Stern D. F., Sporn M. B. (1985). Type beta transforming growth factor: a bifunctional regulator of cellular growth.. Proc Natl Acad Sci U S A.

[OCR_00871] Roberts A. B., Flanders K. C., Kondaiah P., Thompson N. L., Van Obberghen-Schilling E., Wakefield L., Rossi P., de Crombrugghe B., Heine U., Sporn M. B. (1988). Transforming growth factor beta: biochemistry and roles in embryogenesis, tissue repair and remodeling, and carcinogenesis.. Recent Prog Horm Res.

[OCR_00887] Spence A. M., Sheppard P. C., Davie J. R., Matuo Y., Nishi N., McKeehan W. L., Dodd J. G., Matusik R. J. (1989). Regulation of a bifunctional mRNA results in synthesis of secreted and nuclear probasin.. Proc Natl Acad Sci U S A.

[OCR_00893] Terenius L. (1970). Two modes of interaction between oestrogen and anti-oestrogen.. Acta Endocrinol (Copenh).

[OCR_00897] Tucker R. F., Branum E. L., Shipley G. D., Ryan R. J., Moses H. L. (1984). Specific binding to cultured cells of 125I-labeled type beta transforming growth factor from human platelets.. Proc Natl Acad Sci U S A.

[OCR_00903] Yeh H. J., Pierce G. F., Deuel T. F. (1987). Ultrastructural localization of a platelet-derived growth factor/v-sis-related protein(s) in cytoplasm and nucleus of simian sarcoma virus-transformed cells.. Proc Natl Acad Sci U S A.

